# Vascularized Thoracodorsal to Suprascapular Nerve Transfer, a Novel Technique to Restore Shoulder Function in Partial Brachial Plexopathy

**DOI:** 10.3389/fsurg.2016.00017

**Published:** 2016-03-14

**Authors:** Shirley M. Potter, Scott I. Ferris

**Affiliations:** ^1^Victorian Plastic Surgery Unit, St Vincent’s Private Hospital, Melbourne, VIC, Australia; ^2^The Alfred Hospital, Melbourne, VIC, Australia

**Keywords:** brachial plexus injury, thoracodorsal nerve, suprascapular nerve, nerve transfer, vascularized nerve

## Abstract

We describe the clinical outcome of a novel nerve transfer to restore active shoulder motion in upper brachial plexus injury. The thoracodorsal nerve (TDN) was successfully used as a vascularized donor nerve to neurotize to the suprascapular nerve (SSN) in a patient with limited donor nerve availability. At 4 years follow-up, he had regained useful external rotation of the injured limb, with no significant donor site morbidity. Shoulder abduction return was less impressive, however, and reasons for this are discussed. We provide a comprehensive review of the literature on this topic and a subsequent discussion on the details of this novel technique. This is the first reported case of TDN to SSN transfer, and also the first reported case of a vascularized TDN transfer in the English language literature. We advocate direct thoracodorsal to SSN transfer as a valid surgical option for the restoration of shoulder function in patients with partial brachial plexus avulsion, when conventional nerve donors are unavailable.

## Introduction

**Figure 1:**
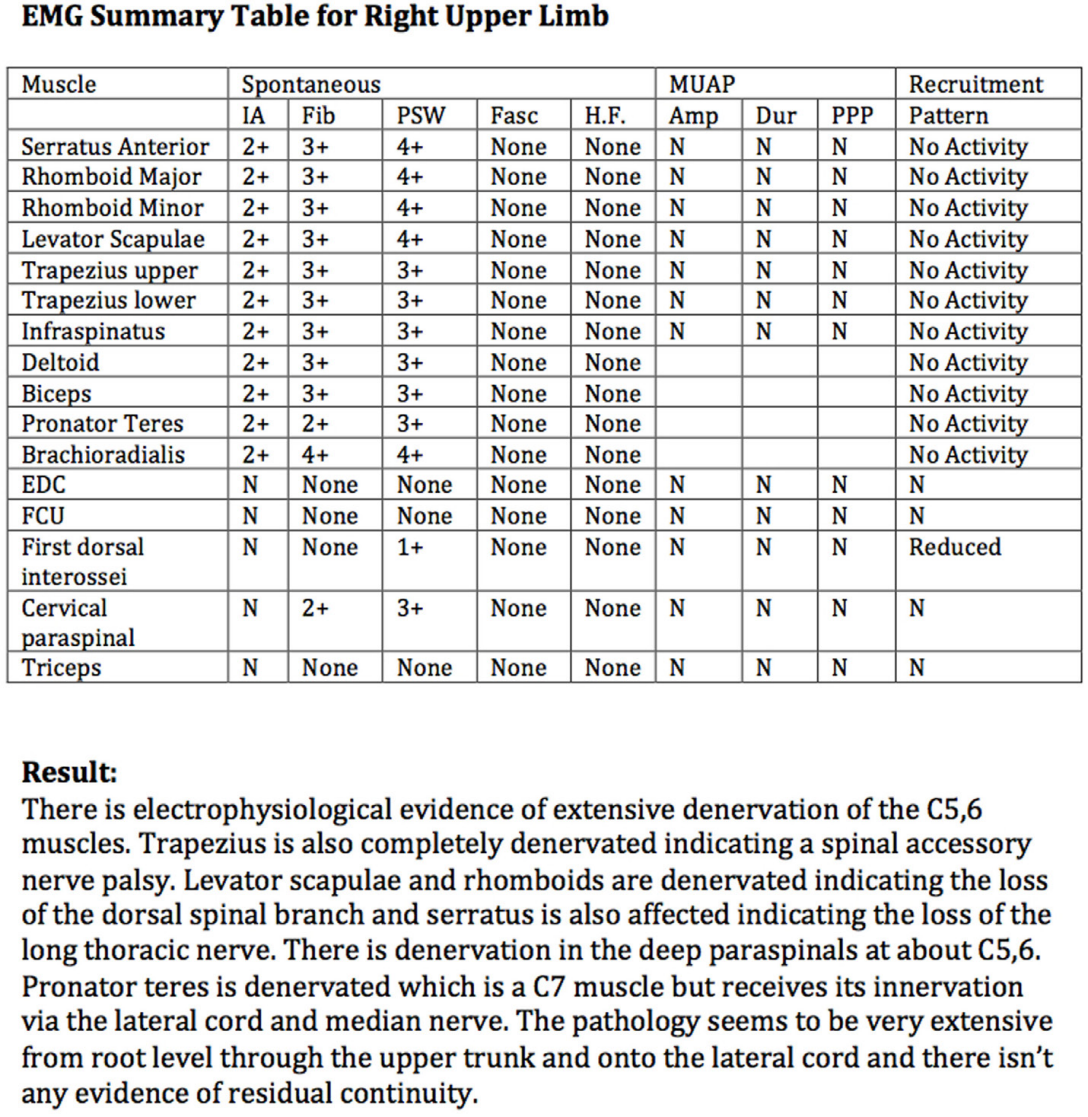
**EMG results table and summary for right upper limb**.

We present the case of a 58-year-old, right-handed truck driver, who sustained a closed right brachial plexus injury following a motor vehicle accident. Clinical examination demonstrated no active shoulder abduction, external rotation, or elbow flexion. Furthermore, he could not shrug his right shoulder. On initial presentation, he had Medical Research Council (MRC) grade 2/5 power in elbow extension; however, this recovered to MRC grade 4 by the time of surgery. Distal motor examination in the hand was normal. These findings suggested an upper plexopathy involving cervical roots 5, 6, ±7, as well as the right spinal accessory nerve (SAN). He had no other significant injuries. Past medical history was non-contributory. He was a non-smoker and a keen right-handed bowls player. Neurophysiological evaluation confirmed denervation of the C5,6 muscles, as well as loss of the SAN, long thoracic nerve, and dorsal scapular nerve (Figure 1).

He underwent surgical exploration at 5 months post injury, which confirmed that both C5 and C6 roots, spinal accessory, long thoracic, dorsal scapular, and phrenic nerves were unavailable as donor nerves for his reconstruction. In order to restore shoulder function, nerve transfer to the suprascapular nerve (SSN) is now a standard procedure. The injury pattern in this case, however, precluded use of some of the commonly used donor nerves (e.g., SAN or cervical roots 5 and 6) for such a nerve transfer. Given this unusual setting and having clinically confirmed a functional latissimus dorsi muscle (LDM) preoperatively, and successfully stimulated the nerve intra-operatively, it was deemed that the thoracodorsal nerve (TDN) was not involved in the injury and, therefore, a vascularized TDN to SSN transfer was performed.

**Figure 2:**
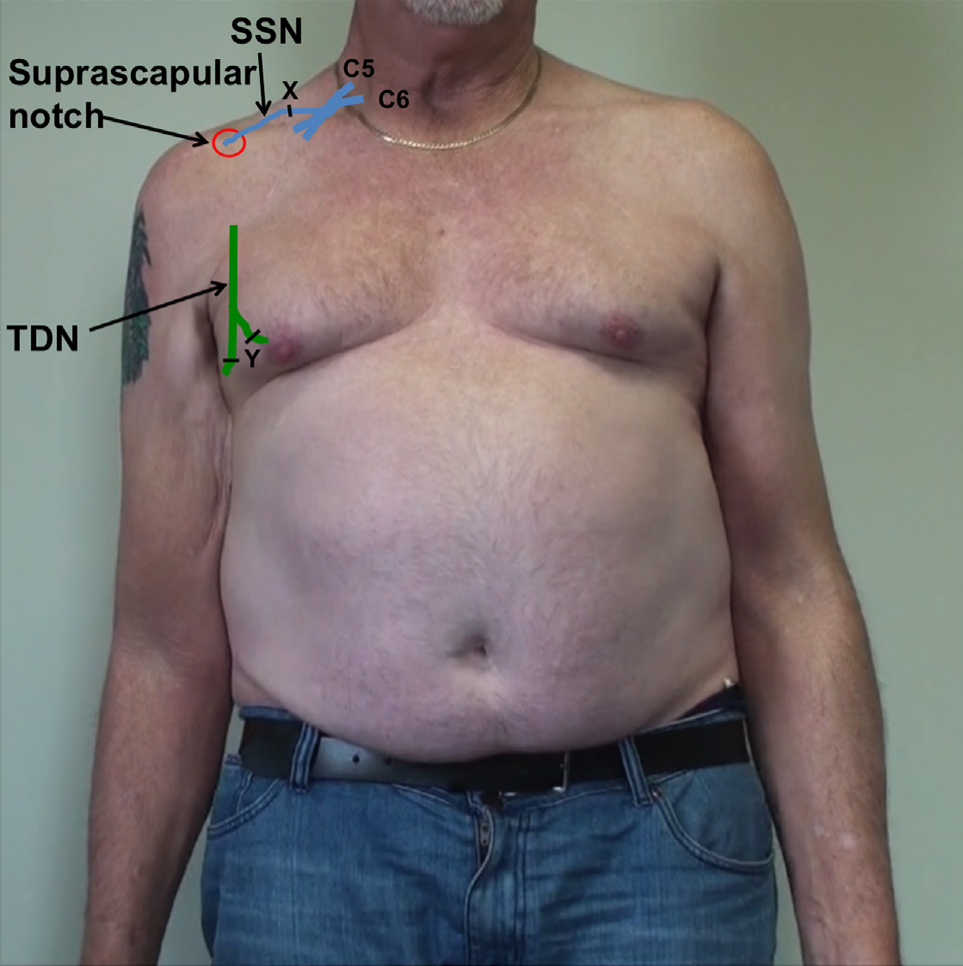
**Schematic representation of the right upper brachial plexus represented in blue, with the suprascapular nerve (SSN) entering the suprascapular notch**. The thoracodorsal nerve (TDN), on the posterolateral border of the axilla, is represented in green. Point X represents the proximal neurotomy on the recipient SSN. Point Y represents the distal neurotomies on the donor thoracodorsal nerves (TDN).

**Figure 3:**
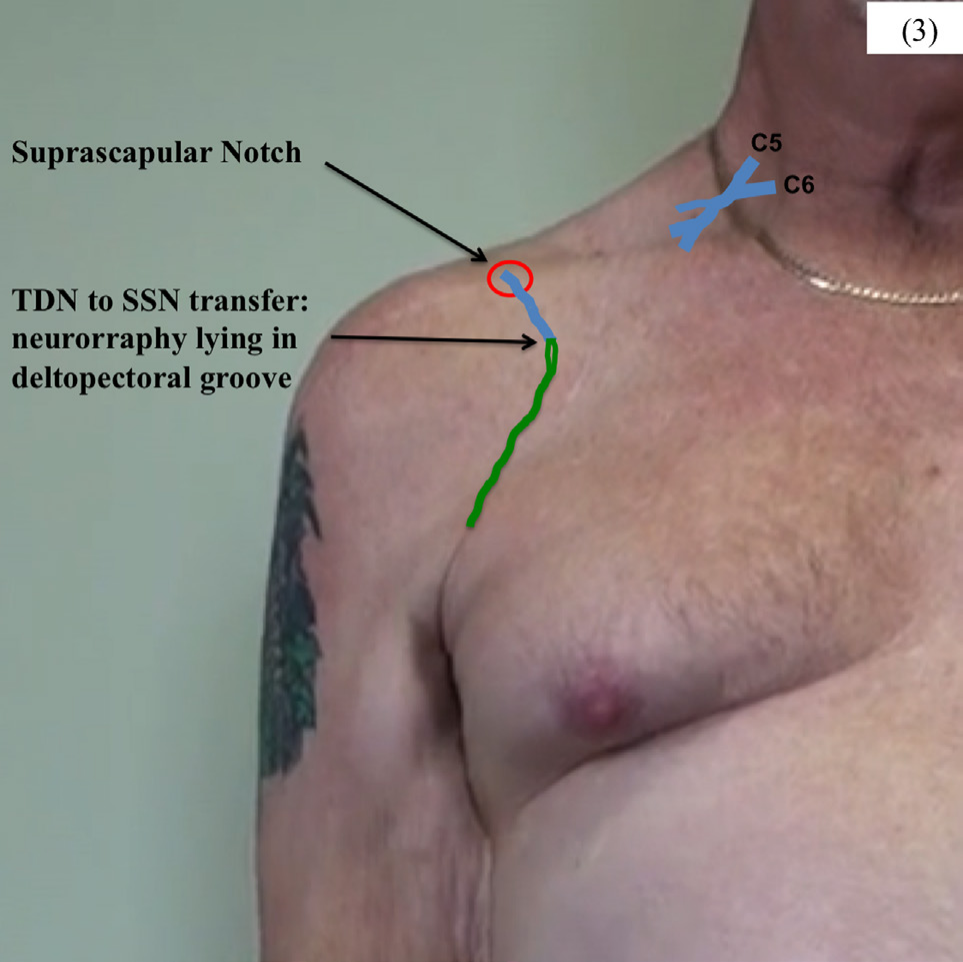
**Neurotomy was performed proximal on the SSN, close to its origin from the upper trunk of the brachial plexus, and the nerve tunneled under the right clavicle**. The terminal branches of the TDN were divided as distal as possible and tunneled under pectoralis major to lie in the deltopectoral groove.

With the patient in a supine position and through a right supraclavicular incision, the SSN was neurolysed from its upper trunk origin to within 2 cm of the suprascapular notch (Figure 2). As expected, the SSN was electrically inactive when stimulated using a handheld nerve stimulator. A proximal neurotomy was performed and the distal nerve transferred deep to the clavicle to lie in the deltopectoral groove. Through a right axillary incision over the palpable border of LDM, the TDN was dissected as a vascularized flap based on the thoracodorsal vascular pedicle. To confirm that the TDN was functional, it was electrically stimulated with a stimulus intensity of 0.5 mA. Neurotomies were performed as distal as possible on the four terminal nerve branches, which were then tunneled deep to pectoralis major muscle toward the recipient nerve (Figure 3). The two longest and largest terminal TDN branches (combined cross-sectional area 4 mm) were repaired to the SSN (cross-sectional area 2.25 mm) in the deltopectoral groove, immediately inferior to the clavicle. The nerve coaptation was approximately 70 mm from the first target muscle. The tension-free neurorrhaphy was performed using standard microsurgical techniques with 9/0 monofilament nylon and fibrin glue around the completed repair. The limb was not splinted but was placed in a shoulder immobilizer for 2 weeks, after which assisted active shoulder and elbow motion were gradually resumed to maintain range of motion. A progressive rehabilitation program, consisting of specific strengthening and coordination exercises, was begun once signs of reinnervation and resumption of muscle activity had appeared.

In addition to the SSN reconstruction, a tri-fascicular (2 × median nerve and 1 × ulnar nerve fascicles) nerve transfer to restore biceps and brachialis muscle function was performed. The apparently recovered triceps nerves were also explored and a triceps nerve to anterior axillary nerve transfer was performed to restore deltoid muscle function.

Six months postoperatively (11 months post injury), and following a rigorous rehabilitation program, he was displaying recovery in the SSN with MRC grade 2 external rotation. At 30 months postoperatively, there was strong infraspinatus action, but lack of supraspinatus function. Given the anterior approach taken in the original nerve transfer procedure, the SSN had not been completely visualized at the suprascapular notch. It was, therefore, decided to explore for second-level injury, release the suprascapular ligament, and neurolyse the SSN. This revealed that the nerve to infraspinatus was of normal caliber, while the nerve to supraspinatus and the common SSN were both enlarged and thickened. These findings offer a possible explanation as to why supraspinatus had not been successfully reanimated by the original TDN to SSN transfer.

At 4 years follow-up, the patient had grade 4 external rotation (active range of motion 0–135°, passive range of motion 0–140°) (Video S1 in Supplementary Material), with palpable and visible infraspinatus contraction. He displayed 20° of active abduction at the shoulder, with MRC grade 2 power. He had grade 4+ elbow flexion through biceps and brachialis action. He had no significant donor site morbidity from any of the three nerve transfers. His Disabilities of the Arm, Shoulder, and Hand (DASH) score was 33, indicating useful functional recovery. He used his right limb to assist with most activities of daily living (e.g., eating, dressing, washing), achieving considerable motor control.

## Background

In the surgical management of brachial plexus injuries, reconstructive priority is given to the restoration of shoulder function and elbow flexion. Compared with elbow flexion restoration, fewer techniques are available for the reconstruction of shoulder abduction and external rotation. Traditional techniques to restore such basic yet critical functions have included nerve grafts or muscle/tendon transfers (1–4). More recently, nerve transfer procedures are increasingly being used, whereby an expendable donor nerve is used to reinnervate a non-functioning, more critical nerve, thereby restoring useful function. Direct nerve transfer provides a closer nerve source to the target muscle using a single coaptation, allowing earlier presentation of regenerating axons to the denervated motor endplates, thereby enhancing the quality, rate, and extent of recovery (5, 6).

## Discussion

To facilitate three-dimensional movement of the limb in space and thereby increase useful function, it is desirable to regain both external rotation and abduction of the shoulder. In their approach to brachial plexus management, it is the authors’ opinion that external rotation is more important than abduction for useful function. This is due to its utility in placing a functioning hand in the working space in front of the body, where most vocational and recreational activities occur (7). The authors acknowledge that abduction is also important, but the hand positions it enables are required less frequently than those provided by external rotation. Many surgeons aim to reinnervate both the suprascapular and axillary nerves when possible, as was attempted in this case. It must be emphasized that the TDN is not the first choice of donor nerve for SSN neurotization. In C5,6,±7 palsies, the preference of the senior author (Scott I. Ferris) is to use the SAN to neurotize the SSN and triceps nerve (when available) to neurotize the axillary nerve. The SAN is the most acceptable and commonly used donor nerve for transfer to the SSN either from an anterior or a posterior approach (8–11); however, it can be concomitantly injured in up to 6–16% of upper plexus injuries (12). Other potential donor nerves include the intercostal nerves (13), phrenic nerve (14), and contralateral SAN (15). Both the SAN and phrenic nerves were unavailable for use in this case. The C5 and 6 roots were also unavailable for grafting. Use of a contralateral nerve requires insertion of a nerve graft, with its inherent disadvantages (6), and can also prove difficult for re-learning given its contralateral origin. The TDN was chosen over an ICN donor because of its higher axon count and perceived ease of retraining, being already integrated into upper limb functions. To our knowledge, this is the first time successful use of this specific nerve transfer has been described.

The TDN is a motor nerve that originates from the posterior cord and remains functional in the majority of upper brachial plexus palsies. Its most frequent pattern of innervation is from C7 to C8 (60%), C7 being the most important, although sometimes it originates from C6 to C8 (16, 17). Innervating the LDM, this is a voluntary muscular nerve with cerebral centers already integrated into the function of the upper extremity. Here, the entire TDN was dissected as distally as possible in order to obtain adequate length for a tension-free direct coaptation with the SSN as close to the target muscle as possible. The mean surgically useable length of the TDN is 12.3 cm (range 8.5–19.0 cm), and the diameter ranges from 2.1 to 3.0 mm (18). Generally, the quality of motor recovery depends on the number of motor axons reinnervating the target muscle (19). The number of myelinated fibers in the TDN ranges from 1530 to 2470, which compares favorably with other more commonly used donor nerves (intercostal nerves: 500–700 axons, SAN: 1700 axons, phrenic nerve: 800 axons) (11, 20, 21). Being a purely motor nerve, there is also no significant axonal mixing. According to these characteristics, the TDN can be considered as an excellent donor in motor nerve transfers.

The TDN was first used as a donor nerve for nerve transfer in brachial plexus palsy by Foerster in 1929 (22), as further reported by Narakas, who employed the nerve when repairing axillary nerve lesions in two cases (23). Since then, TDN transfer has been successfully used to reinnervate the nerve to triceps muscle (24), the musculocutaneous nerve (25, 26), and the long thoracic nerve (18), with no significant donor deficit (18, 24–26). The most successful results have been obtained with TDN transfer to restore elbow flexion. Richardson (27) obtained functional recovery of biceps muscle using the TDN as a donor in four cases with nerve repair delayed for 2 years, while Novak et al. (26) reported successful reinnervation of the biceps muscle in all six cases, and MRC 4 or 5 grades of elbow flexion in five of the six patients. The TDN has never previously been used to neurotize the SSN, making it difficult to provide comparison for the results obtained here. In this case, the patient regained MRC grade 4 external rotation of his shoulder, a similar result to that achieved with conventional donor nerves for SSN neurotization (28, 29).

The advantages of using the TDN as a donor nerve in this case, where other conventional donor nerves were unavailable, were its high axon count (20) and the length of nerve available meant a nerve graft was not required and that the repair was tension-free. It is considered an upper limb nerve and, therefore, already integrated into upper limb functions. The disadvantages of using the TDN to reanimate the shoulder may include its potential antagonistic action to certain limb functions as well as potential donor site morbidity, although neither was a significant problem in this case.

In our patient, TDN transfer for shoulder reanimation produced no significant donor deficits, which is consistent with previous reports in the literature (18, 30). The major function of the LDM is to adduct the upper limb or raise the entire trunk in brachiation (31), as well as assisting in respiration. We believe that loss of these functions following TDN transfer presents an acceptable sacrifice. Some authors suggest that using only one part of the LDM may prevent notable loss of function of the muscle (18). In this case, the entire TDN was sacrificed that denervated the entire LDM. An incompletely denervated LDM could contract and adduct the shoulder when the patient abducts and externally rotates, potentially creating antagonistic co-contraction in this case. This patient had no specific problems during rehabilitation in terms of the antagonistic effects of the LDM.

It should be emphasized that in the majority of cases with extended upper brachial plexus palsy involving the C7 spinal nerve, the TDN is not functional and cannot be used for nerve transfer. It must also be acknowledged that, using the TDN in this way, the denervated LDM cannot then be used for other secondary procedures in cases of unsuccessful nerve repair, i.e., late elbow flexion or shoulder external rotation restoration by latissimus dorsi tendon transfer. We prefer the concept of TDN to SSN transfer to reanimate native muscle rather than transfer of the LDM itself to restore similar function. In general, timely nerve transfers produce better results than muscle transfers, as target muscle biomechanics are not altered (5, 32). Finally, if using the TDN as a donor for nerve transfers, a vascularized nerve transfer may be considered, as doing so adds no additional morbidity. Vascularized TDN transfer was first cited in the Chinese literature by Yu et al. who used it to neurotize the axillary nerve in a series of 10 patients post iatrogenic cervical root injury, resulting in return of at least MRC grade 3 deltoid function (33). Here, we describe vascularized TDN transfer for the first time in the English language literature. The aim of vascularized nerve transfers is to avoid central necrosis due to poor revascularization when large diameter nerves are taken (34, 35). Taylor clinically demonstrated that medium-sized trunk grafts, which could normally undergo central necrosis, could be transferred as vascularized nerve grafts and survive (36). Vascularization allows a nerve graft to avoid the initial period of ischemia and ensures continuous nutrition of the nerve. In experimental studies on vascularized nerve grafts, intraneural fibrosis is avoided and axonal regeneration and target connectivity is enhanced (35). It must be acknowledged that transfer of the thoracodorsal vascular pedicle with the TDN, although demonstrated in this case of nerve transfer, is not completely analogous to the extensive literature on vascularized nerve grafts. Also, it should be emphasized that transfer of the vascular pedicle with the TDN is not mandatory to its use as a donor nerve.

With this novel nerve transfer, we achieved success in restoration of external rotation of the shoulder with excellent range of motion (Video S1 in Supplementary Material) and power. Failure to restore significant shoulder abduction was attributable to pathology at the suprascapular notch, distal to the site of nerve repair. This is a deficiency of the anterior approach taken to the SSN, and not the vascularized TDN donor itself. The preference of the senior author is a posterior approach to access the SSN for nerve transfer, as injury at the level of the suprascapular notch can be evaluated, and this approach allows nerve repair close to the target muscle. In this case, an anterior approach was used as the length of donor nerve was insufficient to reach the suprascapular notch. It is also noteworthy that in this patient there was poor reanimation of deltoid muscle due to triceps nerve involvement in the original injury pattern. In light of our preliminary findings, we believe that direct TDN transfer is a safe and effective surgical option for the restoration of shoulder function in patients with a partial BPI and a functional LDM in the challenging setting of limited donor nerve availability.

## Concluding Remarks

The anatomical diagnosis of the injury, the deficit to be reconstructed, and the available functioning donors are the most important considerations when planning nerve transfer surgeries. The TDN is an ideal donor for motor nerve transfer because of its length and its large number of myelinated fibers. It has previously produced excellent results when transferred to several recipient nerves. Here, we describe, for the first time, its use as a donor nerve for transfer to the SSN. The outcomes achieved in our case indicate that direct TDN transfer is a valid surgical procedure for the restoration of shoulder reanimation in patients with partial BPI in the setting of limited donor nerve availability.

## Consent

The subject gave written informed consent in accordance with the Declaration of Helsinki.

## Author Contributions

All authors made substantial contributions to the conception, design, and completion of the work and have approved the final manuscript.

## Conflict of Interest Statement

The authors declare that the research was conducted in the absence of any commercial or financial relationships that could be construed as a potential conflict of interest. The handling Editor declared a past co-authorship with the author FS and states that the process nevertheless met the standards of a fair and objective review.
